# Mitochondrial integrity modulates mTOR signaling and podocyte function

**DOI:** 10.1016/j.isci.2025.114279

**Published:** 2025-12-05

**Authors:** Cem Özel, Khawla Abualia, Duc Nguyen-Minh, Mahsa Matin, David Unnersjö-Jess, Martin Höhne, Wilhelm Bloch, Henning Hagmann, Richard J.M. Coward, Sebastian Brähler, Bernhard Schermer, Thomas Benzing, Philipp Antczak, Paul T. Brinkkötter

**Affiliations:** 1 Department II of Internal Medicine and Center for Molecular Medicine Cologne, Faculty of Medicine and University Hospital Cologne, University of Cologne, Cologne, Germany; 2 Department of Molecular and Cellular Sport Medicine, Institute of Cardiovascular Research and Sport Medicine, German Sport University Cologne, Cologne, Germany; 3 Bristol Renal, Bristol Medical School, Faculty of Health Sciences, University of Bristol, Bristol, UK; 4 Cluster of Excellence Cellular Stress Response in Aging-associated Diseases (CECAD), Faculty of Medicine and University Hospital Cologne, University of Cologne, Cologne, Germany

**Keywords:** cell biology

## Abstract

Mitochondrial dysfunction has emerged as a key contributor to the pathogenesis of steroid-resistant nephrotic syndrome (SRNS) and genetic focal-segmental glomerulosclerosis (FSGS). This study explores the role of mitochondrial integrity in podocyte biology, focusing on the impact of OMA1, a critical regulator of mitochondrial morphology. Using a model of disrupted mitochondrial homeostasis, we show that mitochondrial dysfunction sensitizes podocytes to insulin, triggering the overactivation of mTOR signaling. Disruption of OMA1 function was achieved through the deletion of *Oma1* or a podocyte-specific knockout of its regulator *Phb2*. Remarkably, simultaneous *Oma1* deletion extended the lifespan of severely affected *Phb2*^pko^ mice, alleviated proteinuria, and restored mitochondrial morphology. Increased mTOR activity was observed in *Phb2*^*pko*^, *Oma1*^*del*^, and *Phb2/Oma1* double-knockout mice. Our findings highlight the critical role of mitochondrial integrity in podocyte function and disease mitigation, providing potential therapeutic insights for mitochondrial dysfunction-associated nephropathies.

## Highlights

• *Oma1* ablation rescues glomerular disease in PHB2-deficient mice
• Disruption of mitochondrial dynamics sensitizes podocytes to insulin
• mTOR hyperactivity alone does not drive glomerular disease

## Graphical abstract

**Figure:**
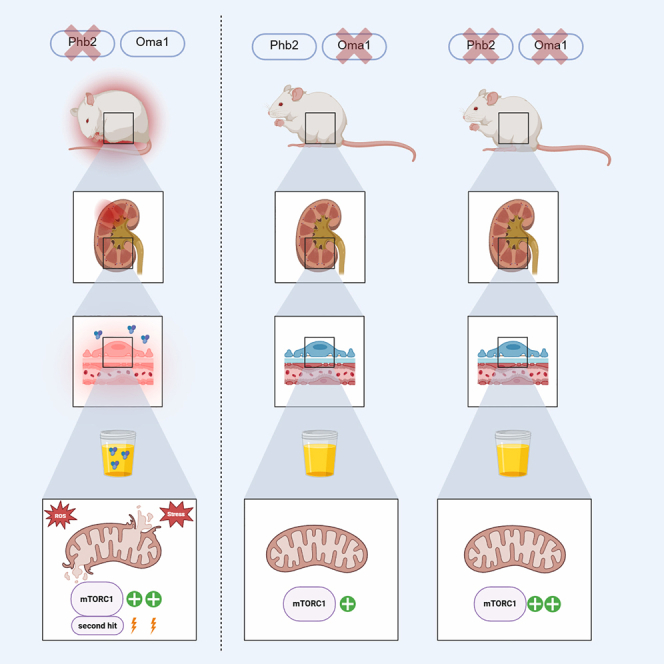

## Introduction

In most cases, kidney diseases evolve from the filtering unit of the blood, the glomeruli.^1^ While damage to all three components of the glomerular filtration barrier – podocytes, endothelial cells, or the glomerular basement membrane – might lead to nephrotic syndrome, most glomerular diseases originate from podocytes.^2^^,^^3^ Many factors, such as genetic mutations, metabolic disorders, or chemical stressors, can contribute to the development of glomerular disease; the accompanying histological pattern of FSGS can be observed in most cases, especially when leading to end-stage renal disease.^4^^,^^5^ To better understand the underlying mechanisms of glomerular diseases, it is essential to consider the cellular processes that influence the function and integrity of podocytes. Mitochondria play a crucial role in this context by orchestrating cellular metabolism and bioenergetics, generating ATP through oxidative phosphorylation, utilizing the tricarboxylic acid (TCA) cycle, and β-oxidation of fatty acids.^6^ Additionally, mitochondria regulate apoptosis, modulate intracellular signaling, and control protein quality.^7^^,^^8^^,^^9^^,^^10^ Dysfunction of mitochondria has been implicated in various diseases and age-related conditions.^11^^,^^12^ To adapt to the changing bioenergetic demands and to maintain their intracellular structure and distribution, mitochondria undergo cycles of fusion and fission, collectively known as “mitochondrial dynamics.”^13^ While mitochondrial fission facilitates apoptosis and mitophagy, mitochondrial fusion allows impaired mitochondria to merge, complementing each other and promoting cell survival.^14^^,^^15^

Previously, we established a link between mitochondrial dysfunction, overactive insulin signaling, and mTOR activation. We showed that podocyte-specific *Phb2* knockout mice exhibit reduced lifespan, proteinuric kidney failure, and increased the phosphorylation of mTOR downstream effector ribosomal protein S6 (RPS6).^16^ Inhibition of mTOR with rapamycin or simultaneous genetic deletion of both insulin and insulin growth factor 1 (*Igf1*) receptors alleviated RPS6 hyperphosphorylation, mitigated renal disease, and extended lifespan, suggesting that altered metabolic signaling plays a significant role in glomerular disease development and progression.^16^ Prohibitins, specifically Prohibitin 1 (PHB1) and Prohibitin 2 (PHB2), are not primarily involved in respiratory chain assembly or function and reside in the inner mitochondrial membrane, forming multimeric ring complexes that stabilize the inner mitochondrial membrane and create functional lipid microcompartments within it.^17^ OMA1, a key peptidase whose activity is regulated by prohibitins, is encapsulated within these lipid microcompartments. As a result of cellular stress, these lipid microcompartments open, releasing OMA1, which in turn cleaves OPA1 into shorter isoforms, S-OPA1, promoting mitochondrial fission.^18^^,^^19^^,^^20^^,^^21^

To deepen our understanding of the relationship between mitochondrial dysfunction and glomerular disease, we used *Phb2*-deficient mice as a model to study mitochondrial disease.^2^^,^^22^ Specifically, we investigated the role of OMA1-mediated mitochondrial processing in podocyte biology *in vivo* by disrupting OMA1 function through podocyte-specific *Phb2* knockout, with and without the additional depletion of OMA1. Additionally, we isolated *Oma1*^del^ podocytes and generated *Phb2*^kd^ podocytes for comprehensive *in vitro* immunoblot studies following insulin treatment. In summary, this study investigated the effects of disrupted mitochondrial integrity on the metabolic signaling of podocytes, aiming to elucidate the poorly understood mechanisms underlying its contribution to glomerular diseases.

## Results

### *Oma1* ablation ameliorates nephrotic syndrome and enhances the survival of podocyte-specific prohibitin 2 knockout mice

**Figure 1:**
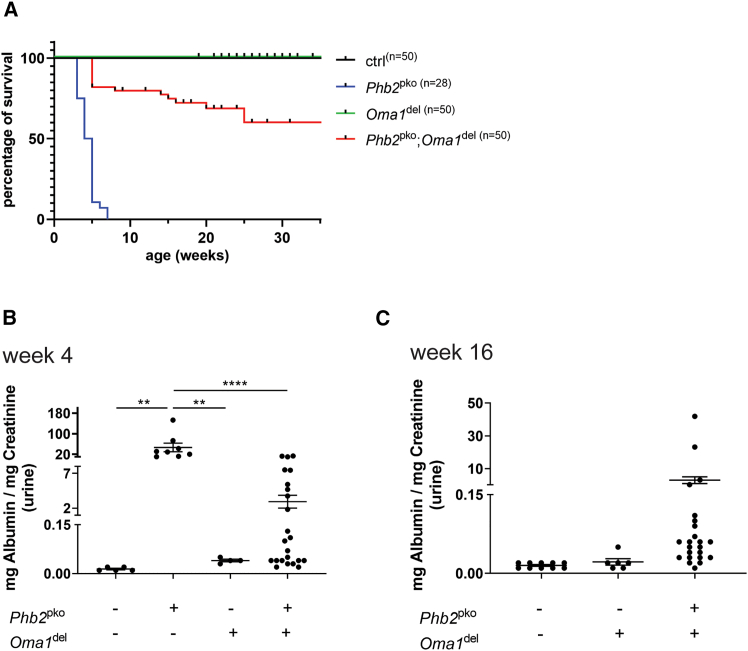
*Oma1* ablation ameliorates nephrotic syndrome and enhances the survival of podocyte-specific *Phb2* knockout mice (A and B) Kaplan-Meier survival curve. *Phb2*^fl/fl^; Oma1^wt/wt^; Nphs2-Cre^wt/wt^ mice served as ctrl (B) urinary albumin/creatinine ratio at week 4. Data are presented as mean ± SEM. ∗*p* < 0.05; ∗∗*p* < 0.01; ∗∗∗*p* < 0.001; ∗∗∗∗*p* < 0.0001. Statistical analysis was performed using one-way ANOVA followed by Tukey’s multiple comparison test. All pairwise group comparisons were tested, and only significant differences are indicated by asterisks. Sample size: ctrl *n* = 5; *Phb2*^pko^*n* = 8; *Oma1*^del^*n* = 4; *Phb2*^pko^; *Oma1*^del^*n* = 23. (C) Urinary albumin/creatinine ratio at week 16. Data are presented as mean ± SEM. ∗*p* < 0.05; ∗∗*p* < 0.01; ∗∗∗*p* < 0.001; ∗∗∗∗*p* < 0.0001. Statistical analysis was performed using one-way ANOVA followed by Tukey’s multiple comparison test. All pairwise group comparisons were tested, and only significant differences are indicated by asterisks. Sample size: ctrl *n* = 10; *Oma1*^del^*n* = 6; *Phb2*^pko^; *Oma1*^del^*n* = 23.

**Figure 2:**
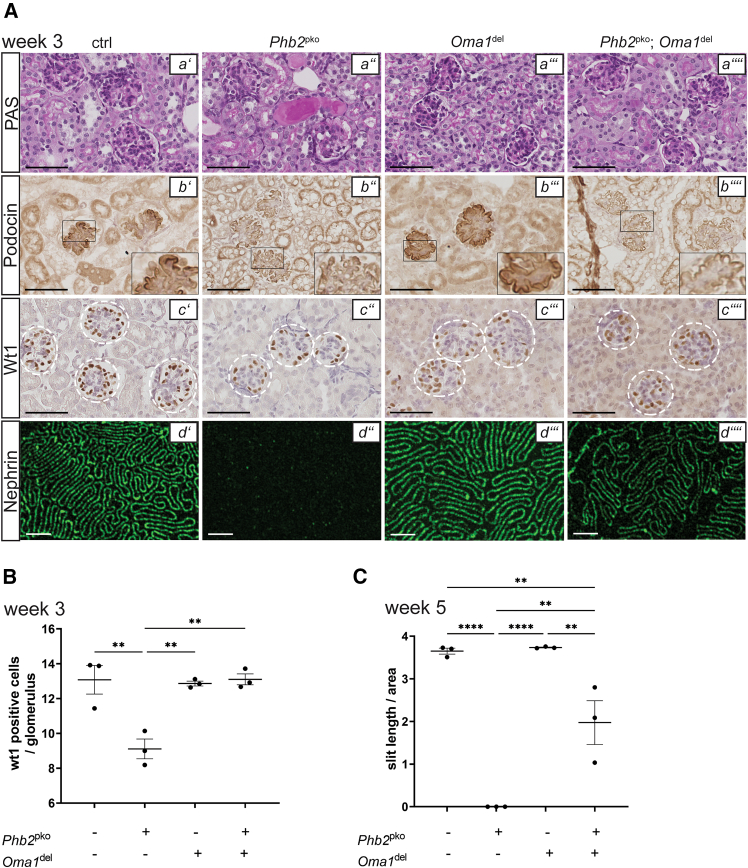
*Oma1* ablation enhances podocyte survival and slit-diaphragm length of podocyte-specific *Phb2* knockout mice (A) Representative periodic acid-Schiff (PAS; a' – a'''') and immunostainings (IHC; Podocin b' – b''''; Wilms Tumor 1 (WT1) c' – c'''') at three weeks (40x magnification, scale bars = 20 μm). STED microscopy at five weeks (d' – d''''; scale bars = 2 μm). Images are representative of *n* = 3 animals per group. (B) Average number of WT1 positive cells per glomerulus at three weeks. Data are presented as mean ± SEM. ∗*p* < 0.05; ∗∗*p* < 0.01; ∗∗∗*p* < 0.001; ∗∗∗∗*p* < 0.0001. Statistical analysis was performed using one-way ANOVA followed by Tukey’s multiple comparison test. All pairwise group comparisons were tested, and only significant differences are indicated by asterisks. *n* = 3 for all experimental groups; >20 glomeruli per mouse were assessed manually. (C) Average slit diaphragm length measured semiautomatically in Stimulated Emission Depletion microscopy (STED) images stained for nephrin at five weeks. Data are presented as mean ± SEM. ∗*p* < 0.05; ∗∗*p* < 0.01; ∗∗∗*p* < 0.001; ∗∗∗∗*p* < 0.0001. Statistical analysis was performed using one-way ANOVA followed by Tukey’s multiple comparison test. All pairwise group comparisons were tested, and only significant differences are indicated by asterisks. *n* = 3 for all experimental groups.

**Figure 3:**
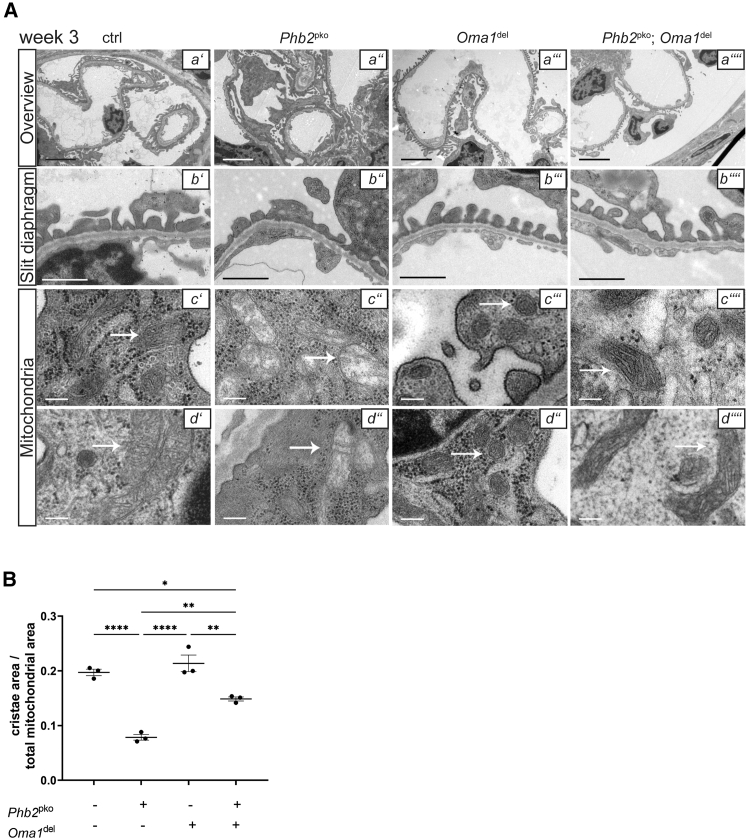
*Oma1* ablation rescues slit diaphragm organization and mitochondrial morphology of *Phb2*^pko^ mice (A) Transmission electron microscopy (TEM) of glomeruli (a' – a''''; 4000x magnification, scale bars = 1 μm), slit diaphragm (b' – b''''; 12,000x magnification, scale bars = 500 nm) and podocyte mitochondria (c' – c'''', d' – d''''; 30,000x magnification, scale bars = 100 nm; marked by arrows). TEM images are representative of *n* = 3 animals per group. (B) Cristae area/total mitochondrial area ratio measured in TEM images. Data are presented as mean ± SEM. ∗*p* < 0.05; ∗∗*p* < 0.01; ∗∗∗*p* < 0.001; ∗∗∗∗*p* < 0.0001. Statistical analysis was performed using one-way ANOVA followed by Tukey’s multiple comparison test. All pairwise group comparisons were tested, and only significant differences are indicated by asterisks. *n* = 3 for all experimental groups (5 mitochondria each).

*Oma1*^del^ mice exhibited normal kidney function and lifespan, while *Phb2*^pko^ mice displayed nephrotic-range proteinuria and premature mortality at six weeks (Figures 1A–1C). Simultaneous genetic deletion of *Oma1* in *Phb2*^pko^ mice led to a significant improvement of proteinuria, thereby ameliorating the overall disease phenotype (Figures 1B and 1C). The survival rate of *Oma1* deficient *Phb2*^pko^ mice was markedly improved, with a mortality rate of 40% at 35 weeks (Figure 1A). Histology at three weeks of age revealed FSGS and widespread protein casts in the kidneys of *Phb2*^pko^ mice, whereas these abnormalities were absent in *Oma1*^del^ mice and mice with both *Phb2*^pko^ and *Oma1*^del^ (Figure 2A). The reduced expression of podocin and nephrin in *Phb2*^pko^ mice was also partially restored by simultaneous *Oma1* ablation, leading to an increase in slit diaphragm length (Figures 2A and 2C). Quantification of podocytes through the staining of their specific nuclear marker Wilms Tumor 1 (WT1) showed a significantly lower count in *Phb2*^pko^ mice, which was recovered by *Oma1* ablation (Figures 2A and 2B). *Oma1*^del^ mice exhibited no significant alterations compared to wildtype control in transmission electron microscopy (TEM) (Figures 3A and 3B). In *Phb2*^pko^ mice, TEM revealed profound alterations in mitochondrial morphology. Quantitative analysis confirmed a severe loss of mitochondrial cristae structure in Phb2pko mice compared to controls (Figures 3A and 3B). This loss of cristae structure was significantly, albeit partially, rescued by additional Oma1 deletion (Figures 3A and 3B).

### *Oma1* modulates insulin signaling and mechanistic target of rapamycin hyperactivity of prohibitin 2-deficient podocytes

**Figure 4:**
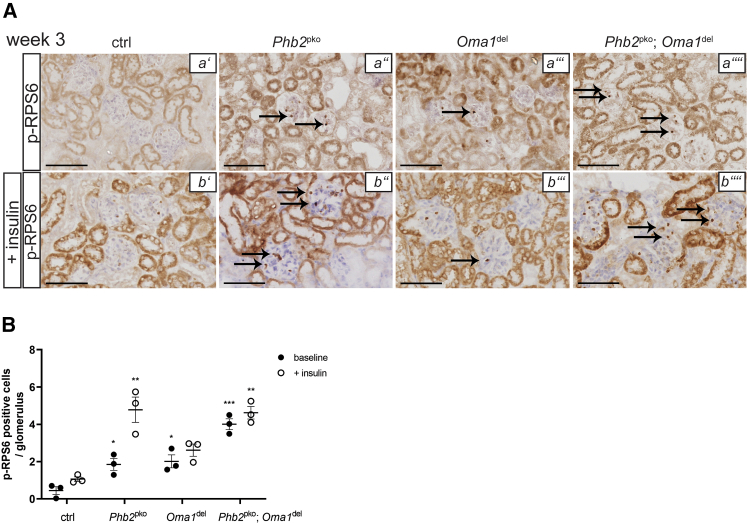
*Oma1* modulates insulin signaling and mTOR hyperactivity of *Phb2*-deficient podocytes (A) Immunohistochemical staining of phosphorylated ribosomal protein S6 (*p*-RPS6) of three week old mice before (a’ – a’’’’) and after (b’ – b’’’’) insulin stimulation (1 IU/kg bodyweight for 60 min). P-RPS6 positive glomerular cells are indicated by arrows in representative images (40x magnification, scale bars = 20 μm). Stainings are representative of *n* = 3 animals per group. (B) Average amounts of *p*-RPS6 positive cells per glomerulus at baseline and after insulin stimulation (1 IU/kg bodyweight for 60 min). Data are presented as mean ± SEM. ∗*p* < 0.05; ∗∗*p* < 0.01; ∗∗∗*p* < 0.001; ∗∗∗∗*p* < 0.0001. Statistical analysis was performed using one-way ANOVA followed by Tukey’s multiple comparison test. All pairwise group comparisons were tested at baseline and after insulin treatment, and only significant differences are indicated by asterisks. *n* = 3 for all experimental groups; >25 glomeruli per mouse were assessed semi-automatically.

To investigate the extent to which the simultaneous loss of *Oma1* in *Phb2* deficient podocytes influences insulin signaling and mTOR activity, we analyzed the phosphorylation of ribosomal protein S6 (RPS6), a downstream target of insulin-dependent mTOR activation in 3-week-old mice, before and after insulin stimulation (1 IU/kg bodyweight for 60 min). Immunohistochemistry revealed that both *Phb2*^pko^ and *Oma1*^del^ mice demonstrated similarly elevated mTOR activity at baseline (Figures 4A and 4B). Following insulin stimulation, only *Phb2*^pko^ mice exhibited a further marked increase in the number of *p*-RPS6-positive glomerular cells (Figures 4A and 4B). In contrast, *Phb2*^pko^; *Oma1*^del^ mice displayed the highest baseline *p*-RPS6 levels, comparable to the insulin-stimulated levels observed in Phb2pko mice (Figures 4A and 4B). Following insulin stimulation, these double knockout mice did not demonstrate a significant additional increase, likely due to a saturation of the signaling pathway (ceiling effect) (Figures 4A and 4B). This suggests a synergistic effect of *Phb2* deficiency and *Oma1* deletion on mTOR hyperactivity (Figures 4A and 4B).

### *Oma1* ablation alters metabolic and kinase signaling in glomeruli

**Figure 5:**
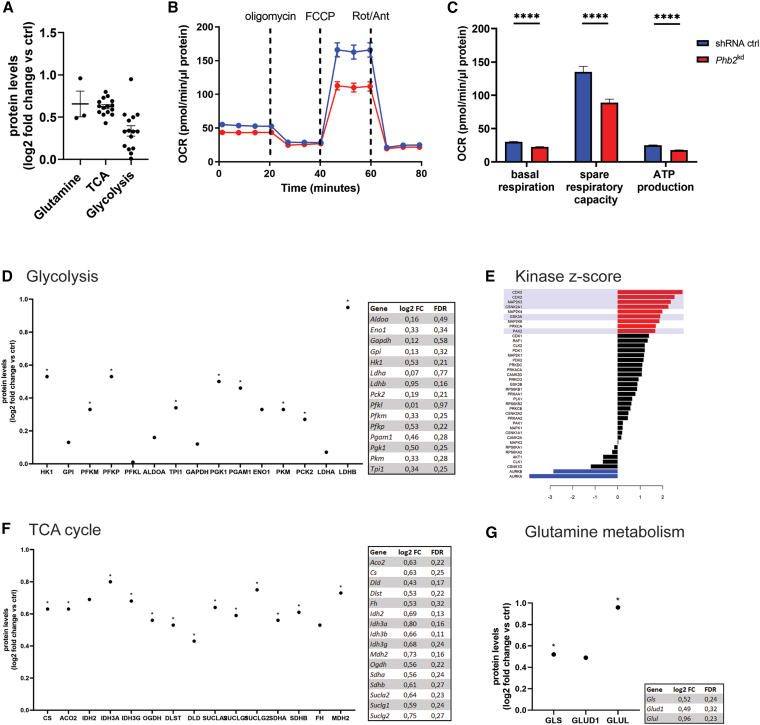
*Oma1* ablation alters metabolic and kinase signaling in glomeruli (A) Overview of expression of proteins associated with glutamine metabolism, the TCA cycle, and glycolysis measured through proteomic studies of glomerular lysates of *Oma1*^del^ mice (log2 fold change vs. ctrl). Data are presented as mean ± SEM. *n* ≥ 3 for all experimental groups. (B–C) Seahorse studies of *Phb2*^kd^ podocytes. Data are presented as mean ± SEM. ∗*p* < 0.05; ∗∗*p* < 0.01; ∗∗∗*p* < 0.001; ∗∗∗∗*p* < 0.0001 (Student’s *t* test). *n* = 3 (3 biological replicates with 17–33 technical replicates per group) (D) Expression of proteins associated with glycolysis in the proteome of *Oma1*^del^ glomerular cells (log2 fold change vs. ctrl). *n* ≥ 3 for all experimental groups. ∗ FDR <0.3. HK1: hexokinase 1, GPI: glucose-6-phosphate isomerase, PFKM: phosphofructokinase (muscle), PFKP: phosphofructokinase (platelet), PFKl: phosphofructokinase (liver), ALDOA: Aldolase (Fructose-Bisphosphate A), TPI1: Triosephosphate Isomerase 1, GAPDH: glyceraldehyde-3-phosphate dehydrogenase, PGK1: phosphoglycerate kinase 1, PGAM1: phosphoglycerate mutase 1, ENO1: enolase 1, PKM: pyruvate kinase M1/2, PCK2: phosphoenolpyruvate carboxykinase 2 (mitochondrial), LDHA: lactate dehydrogenase a, LDHB: lactate dehydrogenase b (E) kinase z-scores of the phosphoproteome of glomerular cells of *Oma1*^del^ mice. *n* = 4 for all experimental groups. (F) Expression of proteins associated with the TCA cycle in the proteome of *Oma1*^del^ glomerular cells (log2 fold change vs. ctrl). *n* ≥ 3 for all experimental groups. ∗ FDR <0.3. CS: citrate synthase, ACO2: aconitase 2, IDH2: mitochondrial isocitrate dehydrogenase [NADP], IDH3A: isocitrate dehydrogenase (NAD(+)) 3 catalytic subunit alpha, IDH3G: isocitrate dehydrogenase (NAD(+)) 3 non-catalytic subunit gamma, OGDH: oxoglutarate dehydrogenase, DLST: dihydrolipoamide s-succinyltransferase, DLD: dihydrolipoamide dehydrogenase, SUCLA2: succinate-CoA ligase ADP-forming subunit beta, SUCLG1: succinate-CoA ligase GDP/ADP-forming subunit alpha, SUCLG2: succinate-CoA ligase GDP/ADP-forming subunit beta, SDHA: succinate dehydrogenase complex flavoprotein subunit a, SDHB: succinate dehydrogenase complex iron sulfur subunit b, FH: fumarate hydratase, MDH2: malate dehydrogenase 2. (G) Expression of proteins associated with glutamine metabolism in the proteome of *Oma1*^del^ glomerular cells (log2 fold change vs. ctrl). *n* ≥ 3 for all experimental groups. ∗ FDR <0.3. GLS: glutaminase, GLUL: glutamine synthetase, GLUD1: glutamate dehydrogenase 1.

To gain deeper insights into intracellular signaling alterations resulting from disrupted mitochondrial integrity, we performed proteomic and phosphoproteomic analyses on glomerular lysates of *Oma1*^del^ mice. Proteomic profiling revealed an increased expression of key proteins associated with glutamine metabolism and the tricarboxylic acid (TCA) cycle (Figures 5A, 5F, and 5G). Additionally, most proteins involved in glycolysis were upregulated in *Oma1*^del^ glomerular cells, with lactate dehydrogenase b upregulated the most (Figures 5A and 5D). Phosphoproteomic analysis identified heightened activity of cyclin-dependent kinases 2 and 5 as well as mitogen-activated protein kinases 3, 4, and 6, suggesting enhanced cellular proliferation and growth, processes commonly associated with mTOR signaling (Figure 5E). Furthermore, we conducted Seahorse studies for the bioenergetic profiling of *Phb2*^*kd*^ podocytes and observed a reduction of spare respiratory capacity, basal respiration, and ATP production (Figure 5B).

### *Oma1* ablation reduces insulin receptor and p38 MAPK phosphorylation in podocytes

**Figure 6:**
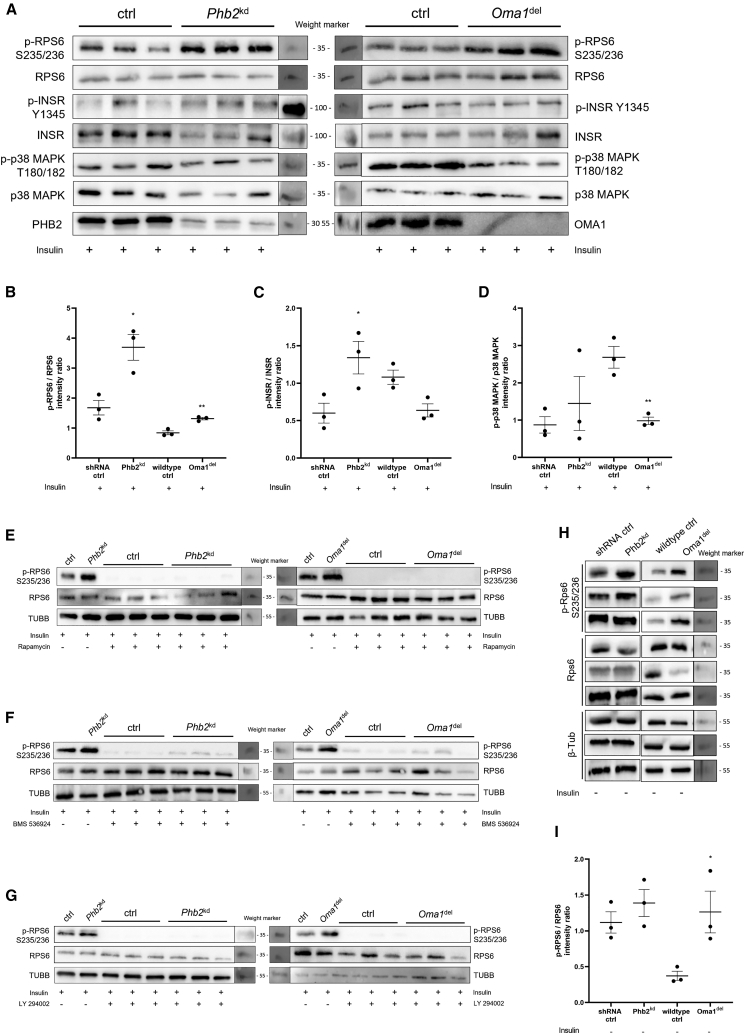
*Oma1* ablation reduces insulin receptor and p38 MAPK phosphorylation in podocytes (A) Immunoblot studies of *Phb2*^kd^ and *Oma1*^del^ podocytes after insulin treatment. *n* = 3 biological replicates per group. (B) Intensity ratio of immunoblot studies of *p*-RPS6 S235/236. Data are presented as mean ± SEM. ∗*p* < 0.05; ∗∗*p* < 0.01; ∗∗∗*p* < 0.001; ∗∗∗∗*p* < 0.0001 (Student’s *t* test versus respective ctrl). *n* = 3 biological replicates per group. (C) Intensity ratio of immunoblot studies of *p*-INSR Y1345. Data are presented as mean ± SEM. ∗*p* < 0.05; ∗∗*p* < 0.01; ∗∗∗*p* < 0.001; ∗∗∗∗*p* < 0.0001 (Student’s *t* test versus respective ctrl). *n* = 3 biological replicates per group. (D) Intensity ratio of immunoblot studies of p-p38-MAPK T180/182. Data are presented as mean ± SEM. ∗*p* < 0.05; ∗∗*p* < 0.01; ∗∗∗*p* < 0.001; ∗∗∗∗*p* < 0.0001 (Student’s *t* test versus respective ctrl). *n* = 3 biological replicates per group. (E) Immunoblot studies of *Phb2*^kd^ and *Oma1*^del^ podocytes after insulin treatment, after mTOR inhibition with Rapamycin. *n* = 3 biological replicates per group. (F) Immunoblot studies of *Phb2*^kd^ and *Oma1*^del^ podocytes after insulin treatment after insulin receptor inhibition with BMS536924. *n* = 3 biological replicates per group. (G) Immunoblot studies of *Phb2*^kd^ and *Oma1*^del^ podocytes after insulin treatment after PI3K/AKT inhibition with LY294002. *n* = 3 biological replicates per group. (H) Immunoblot studies of *Phb2*^kd^ and *Oma1*^del^ podocytes at baseline. *n* = 3 biological replicates per group. (I) Intensity ratio of immunoblot studies of *p*-RPS6 S235/236. Data are presented as mean ± SEM. ∗*p* < 0.05; ∗∗*p* < 0.01; ∗∗∗*p* < 0.001; ∗∗∗∗*p* < 0.0001 (Student’s *t* test versus respective ctrl). *n* = 3 biological replicates per group.

For immunoblot studies, we generated podocytes with an inducible *Phb2* knockdown using a lentiviral approach in heat-sensitive mouse podocytes (HSMPs) and immortalized podocytes isolated from *Oma1*^del^ mice (Figure S2A). Cells were treated with insulin for signaling studies or analyzed at baseline (Figure S2B). At baseline, we observed increased RPS6 phosphorylation in *Oma1*^*de*l^ podocytes and a non-significant trend to increased RPS6 phosphorylation in *Phb2*^*kd*^ podocytes (Figures 6H and 6I). Following insulin treatment, we observed an increased RPS6 phosphorylation *in vitro* in both *Oma1*^*de*l^ and *Phb2*^*kd*^ podocytes (Figures 6A and 6B). Notably, *Phb2*^*kd*^ podocytes exhibited increased Y1345 phosphorylation of the insulin receptor after insulin treatment (Figures 6A and 6C). Furthermore, we observed reduced T180/182 phosphorylation of p38 MAPK in *Oma1*^*del*^ podocytes following insulin-treatment (Figures 6A and 6D–6G), which was also measured in *in vivo* phosphoproteomic studies of *Oma1*^*del*^ mouse glomeruli at baseline (−0,91/−0,85 log2 FC versus ctrl; FDR 0,08/0,07). mTOR hyperactivity could be suppressed in both *Phb2*^*kd*^ and *Oma1*^*del*^ podocytes by inhibiting mTORC1 with rapamycin, insulin receptor autophosphorylation with BMS 536924 or PI3K/AKT with LY294002 (Figures 6E–6G).

## Discussion

In this study, we investigated the relationship between mitochondrial dysfunction and podocyte biology using a mitochondrial disease model with disrupted mitochondrial integrity. Specifically, we explored the role of OMA1 by genetically disrupting its function in combination with a podocyte-specific knockout of *Phb2*, an upstream regulator of OMA1. Our findings demonstrate that genetic *Oma1* ablation rescues glomerular disease phenotypes in podocyte-specific *Phb2*^ko^ mice, resulting in improvements in lifespan, renal function, glomerulosclerosis, podocyte count, slit diaphragm length, slit diaphragm organization, as well as mitochondrial morphology.

Proteomic and phosphoproteomic analyses of *Oma1*^del^ glomeruli reveal the upregulation of key enzymes involved in glycolysis, the TCA cycle, and glutamine metabolism, alongside heightened activity of cell cycle and mitogenic kinases. Enhanced kinase activities suggest the activation of proliferative and repair pathways that may offset mitochondrial damage, while elevated lactate dehydrogenase B (LDHB) expression implies a push toward lactate metabolism, which may enhance cellular resilience against mitochondrial dysfunction. The metabolic shift of podocytes toward glycolysis has been discussed as essential in maintaining energy supply in diabetic conditions, especially in podocyte foot processes.^23^ These metabolic adaptations are particularly crucial for podocyte function, as these highly specialized cells must maintain their complex cytoskeletal architecture and slit diaphragm integrity while having limited regenerative capacity.^2^^,^^24^ Our data show a broad upregulation across glycolysis, the TCA cycle, and glutamine metabolism in Oma1^del^ glomeruli, suggesting an overall enhancement of metabolic capacity. Whether this metabolic profile contributes to podocyte resilience or is merely a consequence of OMA1 deletion requires further investigation. Given that PHB2 inhibits OMA1 and the double-knockout rescues the phenotype, it is plausible that the FSGS phenotype is mediated directly by OMA1 activity rather than a lack of protective metabolic reprogramming. Enhanced glutamine metabolism may support podocyte survival by providing alternative energy sources and maintaining intracellular pH homeostasis, preserving foot process structure and preventing proteinuria.^25^^,^^26^ Furthermore, we show decreased p38 MAPK activation in *Oma1*^del^ podocytes, a kinase that has been implicated as an important positive regulator of stress-induced mTORC1 activation in mammalian and Drosophila tissue culture studies.^27^ Our observed regulatory interplay between p38 MAPK and mTORC1 in *Oma1*^del^ podocytes aligns with recent *in vivo* evidence demonstrating that p38 serves as both a downstream effector and upstream amplifier of mTORC1-driven stress responses.^28^^,^^29^ The attenuation of p38 MAPK signaling may be particularly beneficial in preventing podocyte apoptosis and cytoskeletal derangement, key pathological features that drive glomerulosclerosis progression.^30^ Further studies, including the functional perturbation of the p38 pathway, are needed to assess whether targeting the p38-mTORC1 crosstalk could alleviate glomerular disease in states of mTOR hyperactivation independent of PHB2 deficiency.

Maintaining homeostasis between anabolic and catabolic processes is essential for proper cellular function and requires the precise regulation of metabolic pathways. The mechanistic target of rapamycin (mTOR) plays a critical role in integrating environmental cues, such as nutrient availability, into cellular metabolism. It coordinates diverse cellular processes, including cell growth, differentiation, and autophagy.^31^^,^^32^ Increased mTOR activity has been implicated in several glomerular diseases, such as membranous nephropathy, focal segmental glomerulosclerosis (FSGS), minimal change disease, and diabetic nephropathy.^33^^,^^34^^,^^35^^,^^36^^,^^37^ Conversely, the genetic inhibition of mTOR in podocytes has been associated with glomerular diseases, resulting in early-onset FSGS in rodent models.^38^^,^^39^ In this study, both *Phb2*^pko^ and *Oma1*^del^ mice exhibit elevated baseline mTOR activity, as measured by RPS6 phosphorylation. This finding indicates that OMA1 inhibits mTORC1 activation under basal conditions in podocytes, as loss of OMA1 alone is sufficient to increase mTOR activity. However, only *Phb2*^pko^ mice displayed an amplified response to insulin stimulation. In the double knockout, elevated basal mTORC1 activity might limit further induction by insulin, resulting in an overall response similar to controls. In PHB2 deficient podocytes, the Y1345 hyperphosphorylation of the insulin receptor may potentiate mTORC1 hyperactivity. While the concomitant loss of OMA1 mitigates the overall disease phenotype *in vivo*, whether it directly recalibrates insulin receptor signaling at the level of Y1345 phosphorylation in the context of PHB2 deficiency remains to be determined, as *in vitro* analysis of double-deficient cells was not performed. We acknowledge that the 10 μg/mL insulin concentration used in our *in vitro* experiments is supraphysiological and exceeds standard acute stimulation protocols. This represents a limitation, as it may amplify certain signaling responses. However, the dose was chosen to ensure consistent activation based on established podocyte protocols.^40^^,^^41^ Supraphysiological insulin exposure, as used in our *in vitro* model, can induce receptor and post-receptor desensitization, trigger insulin resistance and non-specific pathway activation (e.g., proliferation, anti-apoptosis, and Akt recruitment).^42^^,^^43^^,^^44^ While such dosing enables robust pathway activation and, in some cases, is a standardized approach to study podocyte biology, it is critical to interpret results within the context of these limitations. As such, extrapolation to physiological scenarios or disease mechanisms must be made cautiously. Importantly, the key genotype-dependent differences we report were robust, but nonetheless, future work should address this limitation with dose-response experiments. Notably, despite the rescue of glomerular disease, mTORC1 hyperactivity was most pronounced in *Phb2*^pko^; *Oma1*^del^ mice at baseline. While mTORC1 activation is often implicated in glomerular pathogenesis, our data strongly suggest that mTORC1 hyperactivity per se is not the primary driver of the disease in this model.^45^^,^^46^^,^^47^ Instead, the rescue conferred by Oma1 deletion indicates that the pathogenic effects of *Phb2* loss are mediated through OMA1 activity. This supports a “two-hit” model: Phb2 deficiency induces mTOR hyperactivity, but requires OMA1-dependent mitochondrial disruption, as evidenced by the quantified loss of cristae structure, to precipitate severe podocyte injury and FSGS. By removing OMA1, the critical second hit is eliminated, allowing podocytes to tolerate elevated mTOR activity. Another contributing factor in the context of *Phb2* deficiency could be an extra-mitochondrial role of *Phb2* in stabilizing the slit diaphragm between adjacent podocyte foot processes, as suggested previously.^48^ Consistent with this view, murine studies in which the podocyte-specific ablation of *Tsc1* led to “super-activation” of mTORC1 show that excessive signaling alone is sufficient to provoke FSGS, whereas more modest, physiologic levels of mTORC1 activity do not cause disease, unless an additional stressor is present.^45^ In that sense, loss of *Phb2* might establish a sensitized background in which extra-mitochondrial slit-diaphragm instability operates as the requisite second hit, jointly with mTORC1 hyperactivity, to precipitate podocyte injury and progressive glomerulopathy. It should be noted, however, that direct evidence for a podocyte-specific “second-hit” mechanism of this kind is currently lacking, and our interpretation represents a conceptual hypothesis based on analogy with other models of kidney disease. Mitochondrial cristae disintegration observed in our Phb2^pko^ mice is a hallmark of bioenergetic failure. Studies in cardiomyocytes have shown that the genetic ablation of PHB2 precipitates a decline in ATP production and a concomitant rise in mitochondrial ROS.^49^ In our Seahorse studies, we could also observe a decrease in ATP production in Phb2^kd^ podocytes. More generally, the inhibition of mitochondrial fusion has been connected to diminished OXPHOS and elevated ROS generation.^50^ These convergent data support the notion that the structural defects we report are sufficient to compromise ATP synthesis and redox homeostasis, providing a link to the mTOR hyper-activation we observe.

These findings highlight the therapeutic potential of targeting OMA1 to modulate mTORC1 signaling in glomerular diseases characterized by mTORC1 hyperactivity. Preclinical studies have already demonstrated promise in targeting OMA1. The peptide SS31, which was associated with decreased levels of OMA1, has alleviated glomerular disease and restored OXPHOS in a mouse model of diabetic kidney disease with mitochondrial dysfunction.^51^ SS31-mediated mitochondrial stabilization has also shown beneficial effects in other rodent disease models, including ischemia-reperfusion, sepsis, ureteral obstruction, and post-ischemic chronic kidney disease.^52^ Similarly, hyperoside, a compound derived from *Abelmoschus manihot* flowers, alleviated adriamycin-induced podocyte injury as well as ischemia/reperfusion-induced acute kidney injury in mice by acting as an OMA1 inhibitor.^53^ In a mouse model of ischemic kidney injury, mitochondrial fragmentation due to OPA1 proteolysis by OMA1 has been implicated as a contributing factor to renal tubular injury and could be rescued by OMA1 ablation.^54^ Our data underscore the potential of OMA1 inhibition as a therapeutic strategy for glomerular diseases and warrant further investigation. Interestingly, the beneficial effects of OMA1 ablation appear to be cell-type specific. In a neuron-specific *Phb2*^nko^ mouse model, additional *Oma1* ablation failed to restore disrupted mitochondrial morphology and prolonged lifespan for less than 10 weeks, with total mortality observed after 30 weeks.^55^

A critical consideration for the translational potential of these findings is the applicability of the *Phb2*^pko^ model. While mutations in PHB2 have not been linked to FSGS in humans, mitochondrial dysfunction is a common feature across various forms of glomerular disease.^56^ In this context, the *Phb2*^pko^ mouse serves as a valuable genetic tool to dissect the consequences of severe mitochondrial stress and OMA1 activation in podocytes. Our findings suggest that targeting OMA1 may offer a therapeutic benefit in a broader spectrum of nephropathies characterized by mitochondrial distress, irrespective of the initiating insult.

In conclusion, our findings indicate that *Oma1* plays a crucial role in modulating podocyte metabolism. Additionally, our findings suggest a positive effect of *Oma1* ablation on mTORC1 signaling, which may contribute to understanding why pharmacological inhibition led to the alleviation of glomerular disease in various rodent models. These results underscore the importance of comprehending the complex interplay between mitochondrial integrity and metabolic signaling in the context of renal health. Further exploration of these pathways may illuminate novel interventions to preserve podocyte function in the context of glomerular diseases linked to mitochondrial disorders.

### Limitations of the study

While our study establishes the role of OMA1 in mitigating glomerular disease in PHB2-deficiency, we did not directly assess OPA1 cleavage in podocytes. As OMA1 is known to cleave multiple substrates, including DELE1, future studies will be necessary to determine the relative contributions of these pathways in podocyte biology and glomerular disease. The glomerular proteomic and phosphoproteomic data derive from bulk lysates containing endothelial and mesangial cells, potentially diluting podocyte-specific signatures. Advanced single-cell proteomics approaches could better resolve compartment-specific metabolic shifts. The complete OMA1 ablation in *Phb2*^pko^ mice contrasts with the partial inhibition achievable pharmacologically. This difference may limit the direct clinical translation of our findings and highlights the need for future studies employing pharmacological or inducible approaches. Notably, *Oma1*^del^ mice exhibit metabolic perturbations in other tissues, complicating the interpretation of systemic versus podocyte-autonomous effects.^57^ In this study, we chose the systemic *Oma1* knockout model primarily due to its immediate availability, which enabled efficient investigation of OMA1 function *in vivo*. Future studies using cell type-specific knockout models and/or single-cell proteomics will be necessary to distinguish these contributions more clearly. The insulin concentration used (10 μg/mL) corresponds to standard podocyte culture protocols but is higher than doses typically applied in acute signaling studies, which may influence pathway activation. While we observed reduced p38 MAPK activity, we did not perform the functional perturbation of p38 to validate its regulatory role in mTORC1 signaling. While our ultrastructural analyses (TEM, STED) revealed marked alterations in mitochondrial morphology and podocyte architecture, the association with mTOR pathway activation was established through parallel signaling analyses. Although these findings suggest a link between disrupted mitochondrial integrity and mTOR hyperactivation, our approach does not directly demonstrate causality or the underlying bioenergetic mechanisms. While our quantitative ultrastructural analyses demonstrate profound changes in mitochondrial morphology (e.g., cristae loss) consistent with altered mitochondrial dynamics, we did not directly measure the rates of mitochondrial fusion and fission events *in vivo*. Therefore, our conclusions relate primarily to mitochondrial integrity and structure rather than the dynamics per se. The proteomic and phosphoproteomic analyses were performed only in Oma1del mice versus controls. To fully understand the metabolic reprogramming that underlies the rescue phenotype in Phb2pko; Oma1del mice, future studies comparing all four genotypes will be necessary.

## Resource availability

### Lead contact

Further information and requests for resources and reagents should be directed to and will be fulfilled by the lead contact, Paul T. Brinkkötter (paul.brinkkoetter@uk-koeln.de)

### Materials availability

This study did not generate new unique reagents.

### Data and code availability

• Proteome and Phosphoproteome data have been deposited to the ProteomeXchange Consortium via the PRIDE partner repository with the dataset identifier PXD071171.
• Original immunoblot images have been deposited at Mendeley Data and are publicly available as of the date of publication at https://doi.org/10.17632/27432g3zhs.1.
• This article does not report original code.
• Any additional information required to reanalyze the data reported in this article is available from the lead contact upon request.

## Acknowledgments

This work was supported by the 10.13039/501100001659Deutsche Forschungsgemeinschaft as part of the Clinical Research Unit KFO329 and Collaborative Research Center TRR422. PB was supported by research funding from the 10.13039/501100001659DFG BR-2955/8 and from the German 10.13039/501100002347Federal Ministry of Education and Research (STOP-FSGS 01GM1901E). CO was supported by Gerok funds of the 10.13039/501100001659Deutsche Forschungsgemeinschaft for a medical research rotation. DNM was supported by the Koeln Fortune Program of the Faculty of Medicine of the 10.13039/501100008001University of Cologne. DUJ was partly supported by the Swedish Kidney Foundation (Njurfonden, F2023-0068, F2022-0051). RJC was supported by the 10.13039/501100000265Medical Research Council (Senior Clinical Fellowship, MR/K010492/1, MR/W019582/1, MR/T002263/1). We thank Ruth Herzog, Vivian Ludwig, and Angelika Köser for excellent technical support and acknowledge the help of the Cologne Excellence Cluster on Cellular Stress Response in Aging-Associated Diseases proteomics and imaging facility. In addition, we would like to express our gratitude to all members of our Lab for helpful discussions and support.

## Author contributions

C.O.: conducted the majority of the experiments, analyzed the data, and wrote the article; K.A.: analyzed the data (Proteomics, Phosphoproteomics); D.N.M.: conducted experiments (immunoblot); M.M: conducted experiments (immunochemistry); D.U.J.: conducted experiments (STED); M.H.: analyzed the data (Fiji Makro for *p*-RPS6 quantification); W.B.: analyzed the data (TEM); H.H.: analyzed the data (Histology, UACR) and funded the study; R.J.M.C.: isolated and immortalized *Oma1*^del^ podocytes; S.B.: analyzed the data (Histology, UACR); B.S.: analyzed the data (Histology, UACR); T.B.: designed the experiments, funded the study, and edited the article; P.A.: analyzed the data (Proteomics, Phosphoproteomics); P.T.B.: designed the experiment, funded the study, analyzed the data, and edited the article; All authors have read, reviewed, and agreed to this version of the article.

## Declaration of interests

The authors declare no competing interests.

## Declaration of generative AI and AI-assisted technologies in the writing process

During the preparation of this work, the author used Perplexity and ChatGPT in order to improve readability. After using this tool, the author reviewed and edited the content as needed and takes full responsibility for the content of the publication.

## STAR★Methods

### Key resources table

**Table:** 

REAGENT or RESOURCE	SOURCE	IDENTIFIER
**Antibodies**		

mouse anti-rabbit secondary antibody	Jackson Immunoresearch	Cat# 315-001-003 RRID: AB_2340031
rabbit anti-podocin	Sigma Aldrich	Cat# P0372 RRID: AB_260918
rabbit anti-phospho-S6 ribosomal protein S235/236	Cell Signalling	Cat# 4858 RRID: AB_916156
rabbit anti-wt1	Santa Cruz	Cat# sc-192 RRID: AB_632611
sheep antinephrin primary antibody	R&D Systems	Cat# AF4269 RRID: AB_2154851
donkey anti-sheep secondary antibody	Thermo Fisher Scientific	Cat # A-21102 RRID: AB_2534723
rabbit anti-phb2	Biolegend	Cat# 611802 RRID: AB_2164497
mouse anti-oma1	Santa Cruz	Cat# sc-515788 RRID: AB_2905488
mouse anti beta-tubulin	Sigma Aldrich	Cat# T0198 RRID: AB_477556
rabbit anti-S6 ribosomal protein	Cell Signalling	Cat# 2217 RRID: AB_331355
phospho Akt S473	Cell Signalling	Cat# 4060 RRID: AB_2315049
rabbit anti-phospho Akt T308	Cell Signalling	Cat# 4056 RRID: AB_331163
rabbit anti-Akt	Cell Signalling	Cat# 9272 RRID: AB_329827
rabbit anti-phospho p44/42 MAPK T202/204	Cell Signalling	Cat# 4370 RRID: AB_2315112
rabbit anti-p42/44 MAPK	Cell Signalling	Cat# 9102 RRID: AB_330744
rabbit anti-phospho p38 MAPK T180/182	Cell Signalling	Cat# 4631 RRID: AB_2139682
rabbit anti-p38 MAPK	Cell Signalling	Cat# 9212 RRID: AB_330713
rabbit anti-phospho insulin receptor Y1345	Cell Signalling	Cat# 3026 RRID: AB_331260
rabbit anti-Insulin receptor β	Cell Signalling	Cat# 3020 RRID: AB_2280418
rabbit anti-phospho GSK3β	Cell Signalling	Cat# 5558 RRID: AB_10694055
rabbit anti-GSK3β	Cell Signalling	Cat# 12456 RRID: AB_2636978

**Bacterial and virus strains**		

pLenti4 / TO (PHB2- hp; Mir neg shRNA)	Laboratory of Paul Brinkkötter	N/A

**Chemicals, peptides, and recombinant proteins**		

OneTaq 2x Master Mix	New England Biolabs	M0482S
Collagenase II	Thermo Fisher Scientific	17101015
Pronase E	Thermo Fisher Scientific	87785
DNAse I	Thermo Fisher Scientific	EN0521
Dynabeads M-450 tosylactivated	Thermo Fisher Scientific	14013
3,3′-Diaminobenzidin	Sigma Aldrich	#Cat D12384
Hematoxylin	Sigma Aldrich	#Cat H9627
Histomount	National Diagnostics	HS-103
Abberior STAR 635P NHS	Abberior GmbH	ST635-0002-1MG
ITS	Corning	354350
BMS 536924	Tocris	4774
LY 294002	Sigma Aldrich	440202
PD 98059	Tocris	1213
Rapamycin	Sigma Aldrich	R8781
RPMI 1640 Medium	Gibco	11875093
PMSF	Sigma Aldrich	#Cat P7626
PIM	Sigma Aldrich	#Cat 526524
Pierce protease and phosphatase inhibitor cocktail	Thermo Fisher Scientific	78440

**Critical commercial assays**		

ABC Kit	Vector Laboratories	PK-7200
PAS-Kit	Sigma Aldrich	12352106
Epoxy embedding medium kit	Sigma Aldrich	12171500
Mouse Albumin ELISA Kit	Bethyl Laboratories	E99-134
Creatinine Colorimetric Assay	Cyman Chemical	Cay500701-96
BCA Protein Assay Kit	Thermo Fisher Scientific	23225
SuperSignal West Femto ECL	Thermo Fisher Scientific	10391544
Oasis HLB 1cc/30mg columns	Waters	WAT094225
Fe-NTA Phosphopeptide Enrichment Kit	Thermo Fisher Scientific	A32992
Seahorse XFp Cell Mito Stress Test Kit	Agilent	103010-100

**Deposited data**		

Oma1^del^ glomerular lysates (phospho)proteome	PRIDE	PXD071171
Immunoblot studies	Mendeley Data	https://doi.org/10.17632/27432g3zhs.1

**Experimental models: Cell lines**		

Mouse: Oma1^del^ podocytes	Laboratory of Richard Coward	N/A
Mouse: Wildtype podocytes	Laboratory of Richard Coward	N/A
Mouse: HSMP Phb2^kd^	Laboratory of Paul Brinkkötter	N/A
Mouse: HSMP mir neg shRNA	Laboratory of Paul Brinkkötter	N/A

**Experimental models: Organisms/strains**		

Mouse: Oma1^del:^ Oma1^-/-^ (C57BL/6)	Laboratory of Paul Brinkkötter Quiros et al.^57^; Venkitachalam	MGI:5311225
Mouse: Phb2^pko^: Phb2^fl/fl^; NPHS2.Cre (C57BL/6)	Laboratory of Paul Brinkkötter Merkwirth et al.^58^; Nelken Moeller et al.^59^; Obst	MGI:3774976 MGI: 2654486

**Oligonucleotides**		

Primers for Genotyping of Phb2^pko^ mice fwd: ATCGTATTGGTGGCGTGCAGCA rev: CGAGGTCTGGCCCGAATGTCA Nphs2:Cre fwd: ACCCGACGGTCTTTAGGG Nphs2:Cre rev: GCAAACGGACAGAAGCATTT	IDT	–
Primers for Genotyping of Oma1^del^ mice fwd: GAGTGCTGTTTCTCTGGGTGT rev: TGCCCTAAACTGAAGGTGTG	IDT	–

**Recombinant DNA**		

pLenti4/TO (PHB2 shRNA construct)	Ising et al.^16^; McNamee	N/A
pLenti4/TO (Scrambled shRNA control)	Ising et al.^16^; McNamee	N/A

**Software and algorithms**		

MaxQuant Suite 1.5.0.1	Max Planck Institute of Biochemistry	RRID:SCR_014485
Adobe Photoshop	Adobe	RRID:SCR_014199
Adobe Illustrator	Adobe	RRID:SCR_010279
Prism 9	GraphPad	RRID:SCR_002798
Fiji / ImageJ	NIH	RRID: SCR_002285
Agilent Wave	Agilent Technologies	RRID:SCR_024491

### Experimental model and study participant details

#### Mouse models

To generate podocyte-specific *Phb2* knockout mice (*Phb2*^pko^), previously described C57BL6/N mice in which exons 3 and 4 of the *Phb2* gene are flanked by loxP sites (control) were mated with Nphs-Cre mice.^58^^,^^59^ *Oma1* whole body knockout (*Oma1*^del^) mice were generated by deleting exon 2 of the *Oma1* gene in C57BL6/N mice as previously described.^57^ Double knockout mice were generated by mating *Phb2*^pko^ with *Oma1*^del^ mice. DNA from mouse ear tags was extracted by incubating ear tags in 60 μL base solution at 95°C for 30 minutes before adding 100 μL neutralization solution. For genotyping 1 μL of the following 0,1 nM primers were added to 2 μL DNA, 12,5 μL OneTaq 2x Master Mix (New England Biolabs, Ipswitch, MA, USA) and PCR-grade water for a total volume of 25 μL, before performing PCR. *Phb2* = ATCGTATTGGTGGCGTGCAGCA + CGAGGTCTGGCCCGAATGTCA; *Oma1* = GAGTGCTGTTTCTCTGGGTGT + TGCCCTAAACTGAAGGTGTG; Nphs-Cre: ACCCGACGGTCTTTAGGG + GCAAACGGACAGAAGCATTT + beta-globin loading control). Annealing temperature for *Phb2* was set at 62°C, for *Oma1* 57°C and Nphs-Cre 59°C. Insulin stimulation of mice was performed after 6h of fasting following i.p. injection of 1 IU / kg bodyweight of insulin 60 minutes before organ harvest. To obtain tissue samples, mice were anesthetized and intracardially perfused with PBS. Kidneys were extracted and fixated in 4% paraformaldehyde for immunohistochemistry or fixated in 4% paraformaldehyde supplemented with 2% glutaraldehyde and 0,1M sodium cacodylate for electron microscopy. Mouse glomeruli were isolated as described previously.^60^ Briefly, mice were euthanized and their kidneys perfused with HBSS containing magnetic beads. Kidneys were minced and placed in digestion solution containing HBSS, Collagenase II, Pronase E and DNAse I (Thermo Fisher Scientific) for 15 minutes. The solution was sieved through 100 μm cell strainers, centrifuged down and washed with HBSS, before purification using a magnetic particle concentrator. All experiments were performed using drug- and test-naive mice of both sexes, selected solely based on genotype and age as determined by PCR genotyping. Mice were housed under standardized specific pathogen-free conditions (e.g., 22°C, 12h light/dark cycle) and used at the ages indicated in the figures and legends. No additional randomization, blinding, or inclusion/exclusion criteria were applied. All eligible mice were included except in cases of unrelated illness. The influence of sex on experimental outcomes was not specifically analyzed, which may limit the generalizability of the findings. All procedures were approved by the University of Cologne Animal Care Committee and local authorities (LANUV NRW; Reference number 81-02.04.2018.A325) and conducted in accordance with national guidelines.

#### Cell lines

Conditionally immortalized heat-sensitive mouse podocytes (HSMP) expressing two tetracycline-inducible PHB2 shRNA constructs were generated through transduction of wildtype HSMP cells using a lentiviral approach with pLenti4/TO plasmids as published previously, while HSMP cells expressing tetracycline-inducible scrambled shRNA served as controls.^16^ Conditionally immortalized heat-sensitive *Oma1*^del^ podocytes were generated after isolating kidneys of *Oma1*^del^ mice as previously described, while isolated podocytes of wildtype mice served as controls.^61^ The sex of the mouse podocyte cell lines used in this study is not known. Cell lines were not further authenticated beyond genotype and functional characterization. All cell lines used in this study were routinely tested for mycoplasma contamination and confirmed to be negative.

### Method details

#### Immunohistochemistry

Formalin-fixed tissue was cut into 4 μm sections, deparaffinized with xylene (VWR, Darmstadt, DE) and rehydrated with graded ethanol. Antigen was retrieved by pressure-cooking samples in 800ml 1x-Tris-EDTA-Tween pH 9 for 10 minutes. Endogenous peroxidase was blocked with 3% hydrogen peroxidase (Fischar, Saarbrücken, DEU), avidin- and biotin blocking was achieved through incubation of samples in 0,2M Sodium Carbonate and 0,2M Sodium Bicarbonate for 15 minutes. Sections were incubated overnight with primary antibodies diluted in TBS with 1% BSA, before incubation with mouse anti-rabbit secondary antibody (Jackson Immunoresearch, West Grove, PA, USA) diluted in TBS with 1% BSA for 60 minutes. Samples were labelled with an ABC Kit (Vector, Burlingame, CA, USA) and developed with DAB (Sigma-Aldrich, St. Louis, MO, USA) according to manufacturer’s instructions. Following counterstaining with hematoxylin (Sigma-Aldrich, St. Louis, MO, USA), sections were dehydrated with ethanol and mounted with Histomount (National Diagnostics, Atlanta, GA, USA). TBST was used for washing in between steps. Images were acquired using Axiovert 200M microscope or Leica SCN400 Slidescanner. The following primary antibodies were used in this study: rabbit anti-podocin (1:400; Sigma-Aldrich, #P0372), rabbit anti-phospho-S6 ribosomal protein S235/236 (1:400, Cell Signalling, #4858), rabbit anti-wt1 (1:400, Santa Cruz, #sc192). Periodic acid Schiff (PAS) stainings were performed using a PAS-Kit (Sigma-Aldrich, St Louis, MO, USA) according to manufacturer’s instructions. Analyses were performed on at least three mice per genotype.

#### Electron microscopy

Tissue samples were fixated in electron microscopy buffer containing 4% paraformaldehyde, 2% glutaraldehyde and 0,1M sodium cacodylate, pH 7.4 for two weeks at 4°C, fixed with 1% osmium tetroxide in 0.1M cacodylate and dehydrated in graded ethanol. Samples were embedded using the epoxy embedding medium kit according to manufacturer’s instructions (Sigma-Aldrich, St Louis, MO, USA). 30 nm sections cut using Ultracut UCT Ultramicrotome were stained with 1,5% aqueous uranylic acetate and lead citrate and examined with a Zeiss EM902 electron microscope. Images were processed with Adobe Photoshop CS4. Quantitative analysis of mitochondrial morphology was performed using Fiji/ImageJ.^62^ The Cristae Area / Total Mitochondrial Area ratio was determined in a blinded fashion. For each genotype, *N* = 3 mice were analyzed. Per mouse, five distinct mitochondria, each from a different podocyte, were evaluated.

#### STED imaging

Kidney sections were optically cleared and immunolabeled following a previously described protocol.^63^ Briefly, formalin-fixed kidney tissue was first treated with a hydrogel solution (4% acrylamide, 0.25% VA-044 initiator, PBS) at 4°C overnight, followed by a polymerization process for 3 hours at 37°C. The tissue was then sliced into 0.3-mm-thick sections using a vibratome. After overnight incubation in clearing solution at 50°C, samples were washed in PBST (0,1% Triton X) for 10 minutes. For immunolabeling, samples were incubated with the primary antibody at 37°C for 24 hours, washed in PBST, and then exposed to the secondary antibody for 24 hours at 37°C. The samples were then washed with PBST for 10 minutes at 37°C prior to mounting. Nephrin was stained using a sheep antinephrin primary antibody (1:100, R&D Systems, #AF4269) and a donkey anti-sheep secondary antibody (Thermo Fisher Scientific, #AB_2534723) conjugated with Abberior STAR 635P. Conjugation of Abberior STAR 635P to secondary antibodies was carried out in-house as follows. Abberior STAR 635P NHS ester (Abberior GmbH, ST635-0002-1MG) was dissolved at a dilution of 10 mg/ml in DMSO. The conjugation was carried out under basic conditions, by adding 1M NaHCO_3_ at a dilution of 1:10 to 1X PBS. Secondary antibodies and NHS ester was then added at a dye-to-antibody ratio of 20 (20 times molar excess of dye as compared to antibody). Following incubation over one hour at room temperature, excess fluorophores were removed using a centrifugal filter (Sigma-Aldrich, #UFC5030). Centrifugation was repeated after adding PBS with 0,1% sodium azide, which was added again after centrifugation for a final antibody concentration of 1 mg /ml. Cleared and immunolabeled sections were prepared for imaging by immersing them in 80% fructose solution with 0.5% 1-thioglycerol for 1 hour at 37°C and were then mounted on a glass-bottom dish (Mattek #P35G1.5-14-C). Imaging was performed using a Leica SP8 3X STED system. Morphometric parameters were quantified as described previously, using Fiji software with a macro allowing semi-automatic quantification of slit diaphragm length per area.^64^

#### Urinary albumin/creatinine ratio

Urinary Albumin/Creatinine Ratios were determined by ELISA (Mouse Albumin ELISA Kit, Bethyl Laboratories Inc., Montgomery, TX, USA) and Colorimetric Assay (Creatinine Colorimetric Assay Kit, Cayman Chemical, Ann Arbor, MI, USA) according to manufacturer’s instructions.

#### Cell culture

For maintenance, cells were incubated at 33°C. For differentiation, cells were thermoswitched to 37°C for ten days. For insulin treatment 10 μg insulin / ml medium was added for three hours, after cells were treated with FBS-free medium overnight (ITS; Corning, Corning, NY, USA).^40^^,^^41^^,^^65^ For inhibitor studies, the respective inhibitors were added before insulin treatment using the following target concentrations and durations: 1μM BMS 536924 (Tocris, #4774) in DMSO for 60 minutes, 20μM LY 294002 (Merck, #440202) in DMSO for 60 minutes, 10μM PD 98059 (Tocris, #1213) in DMSO for 60 minutes, 10 ng/ml Rapamycin (Sigma, #R8781) for 120 minutes. Podocytes were cultured in RPMI medium supplemented with 10% FBS, 5% 100 mM SPS and 5% 1M Hepes buffer (Gibco / Thermo Fisher Scientific, Waltham, MA, USA). For each measurement, at least three biological replicates were used.

#### Immunoblotting

Cells were scraped and lysed in ice cold lysis buffer (1% Triton-X-100, 50mM TrisHCl ph 7.4, 150mM NaCl, 50mM NaF, 15mM Na_4_P_2_O_7_) supplemented with NaO_3_V and protease/phosphatase inhibitors PMSF and PIM (Sigma-Aldrich, St Louis, MO, USA). Protein concentration of samples were measured using BCA Protein Assay Kit (Thermo Fisher Scientific, Waltham, MA, USA). 30μg of total protein was diluted with Laemmli and boiled at 95°C for 5 minutes. Samples were separated with SDS-PAGE, transferred to PVDF (Millipore) membranes and incubated with primary antibodies overnight at 4°C. Following incubation with HRP-conjugated secondary antibodies for 60 minutes, membranes were incubated in SuperSignal West Femto ECL (Thermo Fisher Scientific, Waltham, MA, USA) before immunoreactive bands were visualized using Fusion Solo. The following antibodies were used for this study: rabbit anti-phb2 (1:1000, Biolegend, #611802), mouse anti-oma1 (1:1000, Santa Cruz, #sc515788), mouse anti beta-tubulin (1:1000, Sigma Aldrich, #T0198, rabbit anti-phospho-S6 ribosomal protein S235/236 (1:1000, Cell Signalling, #4858), rabbit anti-S6 ribosomal protein (1:1000, Cell Signalling, #2217), rabbit anti-phospho Akt S473 (1:1000, Cell Signalling, #4060), rabbit anti-phospho Akt T308 (1:1000, Cell Signalling, #4056), rabbit anti-Akt (1:1000, Cell Signalling, #9272), rabbit anti-phospho p44/42 MAPK T202/204 (1:1000, Cell Signalling, #4370), rabbit anti-p42/44 MAPK (1:1000, Cell Signalling, #9102), rabbit anti-phospho p38 MAPK T180/182 (1:1000, Cell Signalling, #4631), rabbit anti-p38 MAPK (1:1000, Cell Signalling, #9212), rabbit anti-phospho insulin receptor Y1345 (1:1000, Cell Signalling, #3026), rabbit anti-Insulin receptor β (1:1000, Cell Signalling, #3020), rabbit anti-phospho GSK3β (1:1000, Cell Signalling, #5558), rabbit anti-GSK3β (1:1000, Cell Signalling, #12456).

#### nLC-MS/MS

Proteomic and phosphoproteomic studies were conducted as previously described.^60^^,^^66^ Briefly, cells were lysed in lysis buffer containing 8M urea, 50 mM ammonium bicarbonate and Pierce protease and phosphatase inhibitor cocktail (Thermo Fisher Scientific, Waltham, MA, USA). Chromatine was degraded using Athena Ultrasonic Probe Sonicator (Athena Technologies, Mumbai, IND) and cleared using centrifugation (16.000g, 45 minutes at 4°C). After determination of protein concentration with a BCA Protein Assay Kit (Thermo Fisher Scientific, Waltham, MA, USA), 800μg of total protein per sample was reduced with 5 mM DTT for 60 minutes and alkylated with 20 mM Iodoacetamide for 60 minutes before adding 50 mM ammoniumbicarbonate to the solution at a ratio of 1:4 and trypsin for over-night digestion supplemented to the solution at a ratio of 1:100. The following day 100% formic acid was added at a ratio of 1:200 to stop digestion and samples were cleared using centrifugation (16.000g, 10 minutes, room temperature). Purification was performed using Oasis HLB 1cc/30mg columns (Waters Corp., Milford, MA, USA) according to manufacturer’s instructions. Before drying samples using speedvac, 30μg of total protein was stored at -20°C for proteome measurements. Phosphopeptides were enriched using Fe-NTA Phosphopeptide Enrichment Kit with immobilized metal affinity chromatography colums according to manufacturer’s instructions (Thermo Fisher Scientific, Waltham, MA, USA), dried using speedvac and resuspended in 0,1% formic acid. Peptides were analysed as previously described using an LTQ Orbitrap Discovery mass spectrometer (Thermo Fisher Scientific, Waltham, MA, USA) coupled to a Proxeon EASY-nLC II nano-LC system (Proxeon/Thermo Fisher Scientific, Waltham, MA, USA) with a flowrate of 250nl/min over 150 minutes.^60^ MS1 survey scan was acquired from 300-2000 m/z at a resolution of 70000. The 10 most intense peptides were isolated within a 2 Da window and fragmented by higher-energy collisional dissociation fragmentation as previously described.^67^ Four biological replicates were used per experimental group.

#### Seahorse studies

Cellular oxygen consumption rate (OCR) were quantified using a Seahorse XFp Analyzer (Agilent Technologies, Santa Clara, CA, USA). Mitochondrial oxidative phosphorylation (OXPHOS) was assessed using the Seahorse Mito Stress Test Kit, according to the manufacturer’s instructions. Briefly, 40,000 cells per well were seeded in XFp 96-well cell culture plates and allowed to differentiate for ten days. On the day of analysis, culture medium was replaced with Seahorse DMEM (Agilent, #103575) and supplemented with 10 mM glucose, 2 mM glutamate and 1 mM sodium pyruvate. Cells were incubated at 37°C for 45 minutes prior to the assay. During analysis 1 μM oligomycin, 2 μM FCCP, and 1 μM rotenone/antimycin A were sequentially injected into each well. All measurements were normalized to total protein content, determined by BCA Protein Assay (Thermo Fisher Scientific, Waltham, MA, USA). Data analysis and visualization were performed using Agilent Wave software. For statistical analysis, technical replicates (17-33 wells per condition) were averaged for each independent biological experiment (*n* = 3).

### Quantification and statistical analysis

All statistical details of experiments, including the statistical tests used, the exact value of n, what n represents (e.g., number of animals or independent biological replicates) and dispersion measures (mean ± SEM) are reported in the respective figure legends.

#### Bioinformatic analysis (nLC-MS/MS)

Raw files were analysed using MaxQuant Suite 1.5.0.1 algorithms with LFQ and match between runs enabled and searched against mouse uniprot reference database. Mass accuracy was 20ppm with deisotoping option enabled while mass tolerance was 0,5 Da. Fixed modifications were carbamidomethylation of cystines, variable modifications were phosphorylation on S, T, Y and oxidation of methionine with a maximum of four modificatons allowed. Protein, peptide and site false discovery rate was set to be 0.1 by default. LFQ and phosphorylation site intensities were logarithmized, potential contaminants removed and normalized by substraction of the mean. Using Perseus 1.5, GO Terms were annotated and categorical 1D enrichment analysis by a Fisher’s exact test was performed.

#### Visualization and statistical analysis

Adobe Photoshop, Adobe Illustrator and GraphPad Prism 9 were used for visualization. Results are expressed as means ± SEM. Statistical analyses were performed using GraphPad Prism 9. For experiments involving multiple groups, ordinary one-way ANOVA was performed followed by Tukey’s multiple comparison test, assessing all pairwise comparisons between groups. Only statistically significant differences are indicated in the figures by asterisks and non-significant comparisons are not labelled in the graphs for clarity. For comparisons involving two groups, statistical significance was assessed using an unpaired two-tailed Student’s t-test. Significance levels are defined as follows: ∗*P* < 0.05; ∗∗*P* < 0.01; ∗∗∗*P* < 0.001; ∗∗∗∗*P* < 0.0001; for (phospho)proteome studies ∗ FDR < 0.3. No randomization or blinding was performed for *in vitro* experiments.
