# Supplementary material for: Mitochondrial integrity modulates mTOR signaling and podocyte function
Source: iScience. 2025 Dec 5;29(1):114279. doi: 10.1016/j.isci.2025.114279 (PMC12774701; doi:10.1016/j.isci.2025.114279)
Supplement: Document S1. Figures S1 and S2 [file mmc1.pdf]

iScience, Volume *29*

Supplemental information

Mitochondrial integrity modulates

mTOR signaling and podocyte function

Cem Özel, Khawla Abualia, Duc Nguyen-Minh, Mahsa Matin, David Unnersjö-Jess, Martin Höhne, Wilhelm Bloch, Henning Hagmann, Richard J.M. Coward, Sebastian Brähler, Bernhard Schermer, Thomas Benzing, Philipp Antczak, and Paul T. Brinkkötter

Supplementary Figure 1

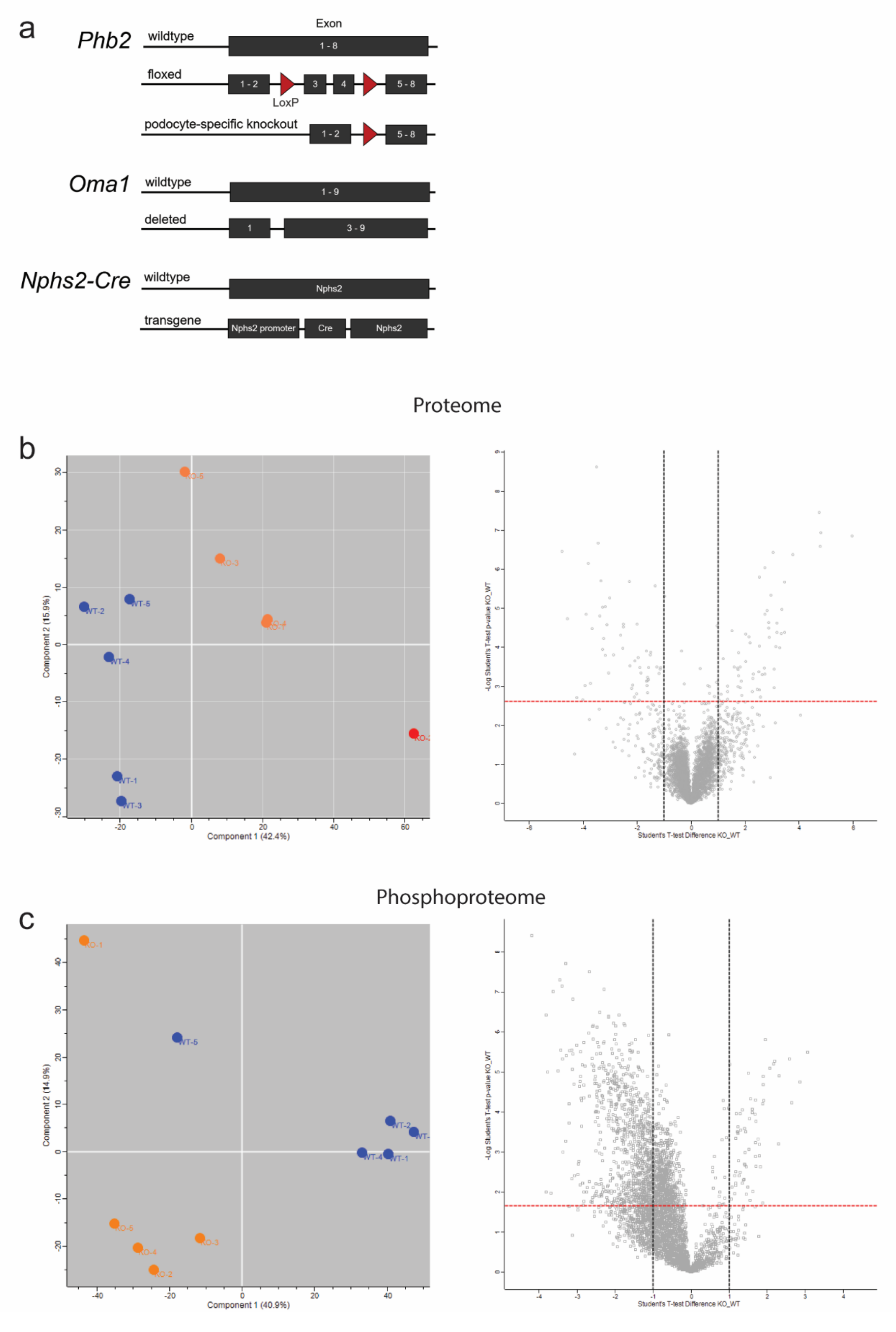

Supplementary Figure 1: mouse genetics and (phospho)proteome quality indicators. Related to Figure 1 and 5

(A) schematic depiction of genetic modifications and wildtype alleles.60 (B) principal component analysis (PCA) and volcano plot of proteome studies of glomerular lysates of Oma1del mice (C) PCA and volcano plot of phosphoproteome studies of glomerular lysates of Oma1del mice

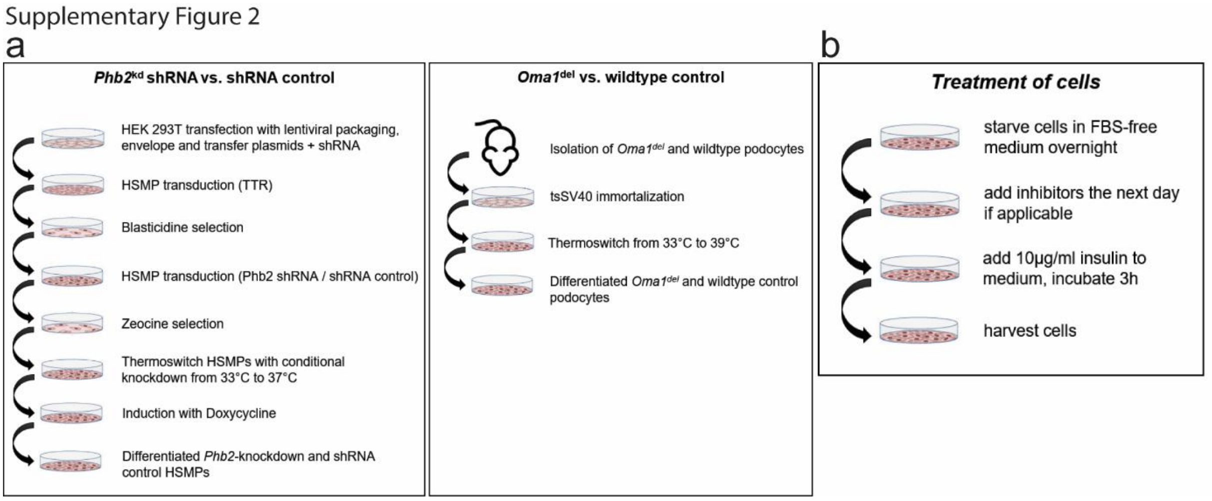

Supplementary Figure 2: Phb2kd and Oma1del podocyte generation, treatment protocol and immunoblot studies. Related to Figure 6.

(A) schematic depiction of the generation of Oma1del podocytes[S1] (B) schematic depiction of the cell culture treatment protocol.

Supplemental References

S1. Xie Y, Li H, Luo X, et al. IBS 2.0: an upgraded illustrator for the visualization of biological sequences. *Nucleic acids research*. Jul 5 2022;50(W1):W420-W426. doi:10.1093/nar/gkac373
